# A Multimodal Sensor-Based Self-Supervised Learning Framework for Low-Noise System State Prediction and Anomaly Detection

**DOI:** 10.3390/s26123851

**Published:** 2026-06-17

**Authors:** Kexin Guo, Jingwen Wang, Jiayu Lin, Ningjing Chen, Hengyuan Chen, Zilang Zhou, Manzhou Li

**Affiliations:** 1 China Agricultural University, Beijing 100083, China; 2 National School of Development, Peking University, Beijing 100871, China

**Keywords:** multimodal sensing, self-supervised representation learning, sensor data fusion, system state prediction, anomaly detection

## Abstract

To address the challenges of strong signal noise, pronounced cross-modal asynchrony, high subjectivity in manually defined state labels, and insufficient model stability under extreme abnormal conditions in multi-source sensor systems, a low-noise system state prediction and anomaly detection method based on multimodal sensor signals and self-supervised representation learning is proposed. Environmental sensing data, device status data, network transmission data, operational behavior data, and event log data are uniformly modeled as system state perception signals. A temporal masking-based state structure modeling method, a state-oriented contrastive learning representation constraint mechanism, and a state representation and downstream prediction task alignment strategy are designed to learn stable, transferable, and interpretable system state features. Experimental results demonstrate that the proposed method achieves the best performance in multimodal sensor state prediction and anomaly detection tasks, with mean squared error (MSE), mean absolute error (MAE), and root mean square error (RMSE) values of 0.0167, 0.0856, and 0.1291, respectively, outperforming baseline models such as GARCH, MLP, LSTM, TCN, and Transformer. Meanwhile, IC, RankIC, and AUC reach 0.494, 0.460, and 0.815, respectively, indicating stronger state-ranking capability and improved discrimination between high-abnormality and low-abnormality states. At the classification recognition level, superior accuracy, precision, recall, and F1-score are also achieved by the proposed method, suggesting that potential abnormal states can be identified more accurately. Ablation experiments verify the effectiveness of multimodal fusion, temporal masking modeling, self-supervised contrastive constraints, and task alignment strategies. Robustness experiments further show that lower prediction errors and higher AUC can still be maintained under high-fluctuation and extreme-shock states, demonstrating strong noise resistance, stability, and practical application potential in complex sensor system scenarios.

## 1. Introduction

System state perception and robust modeling are core scientific issues in intelligent monitoring, industrial equipment management, and environmental monitoring, as they are closely related to system anomaly detection and early warning capability and directly affect the reliability of intelligent control and decision-making systems in complex dynamic environments [1]. With the widespread deployment of Internet of Things devices and the digital transformation of industrial systems, multimodal sensor networks have become a primary means of acquiring system states. Various sensor signals, such as temperature, humidity, vibration, illumination, current, voltage, and environmental parameters, can reflect state changes in equipment and environments at both microscopic and macroscopic levels in real time [2]. Meanwhile, sensor data are characterized by high frequency, multimodality, strong noise, and heterogeneity. They are no longer limited to single physical-variable sequences, but have gradually evolved into complex information systems composed of multiple sensors, environmental indicators, and auxiliary signals [3]. Therefore, methods capable of extracting robust, low-noise, and transferable system state features from high-noise, multimodal, and heterogeneous sensor data are of significant theoretical value and practical importance for intelligent monitoring and anomaly early warning [4].

From the perspective of traditional research paradigms, system states or risks are usually quantified as a single indicator or threshold, based on which supervised learning or statistical modeling tasks are constructed [5]. Early methods mainly relied on signal statistical features, threshold-based stratification, time-series modeling, or rule-based anomaly detection. These methods laid the foundation for system monitoring and improved the standardization of state recognition to some extent [6]. However, most traditional methods are built on assumptions of stationarity, linear relationships, or specific distributions, whereas signals in real systems often exhibit non-stationarity, heavy-tailed distributions, event-driven variations, and strong noise. Consequently, models can easily fail under high-interference conditions, extreme events, and system configuration changes [7]. More importantly, state labels or anomaly annotations are usually not objective quantities that can be directly observed; instead, they are indirectly constructed through specific rules, threshold settings, or expert experience, and are therefore inherently subjective, biased, and time-dependent [8]. This means that models trained on historical labels may over-rely on existing definitions, and performance degradation and insufficient generalization may occur once system mechanisms change or label distributions drift. In addition, in multimodal sensor scenarios, substantial differences among signals in temporal granularity, statistical distribution, and noise structure make effective cross-modal collaborative modeling difficult for traditional methods.

In recent years, deep learning has provided new technical pathways for multimodal sensor time-series analysis [9]. Models such as RNNs, LSTMs, and GRUs model temporal dependencies through recurrent structures and have demonstrated stronger nonlinear fitting capabilities than traditional statistical models in system state prediction, anomaly detection, and fault identification. Transformer and its variants have achieved further breakthroughs in long-range dependency modeling through self-attention mechanisms, promoting the evolution of sensor prediction models toward higher-dimensional and more complex structures [10]. Meanwhile, multimodal fusion learning has attracted increasing attention, and attempts have been made to jointly model signals such as temperature, humidity, vibration, illumination, and current to improve the comprehensiveness of system state perception [11]. However, most existing deep learning methods remain label-intensive supervised paradigms that rely on large-scale, high-quality manual labels and are therefore susceptible to label noise and subjectivity. Moreover, simple multimodal concatenation or static fusion is inadequate for handling practical problems such as asynchronous alignment, cross-scale dependencies, and high-noise interference [12]. In contrast, self-supervised representation learning can mine more essential structural features from unlabeled data through temporal prediction, masked reconstruction, and contrastive learning, thereby providing new possibilities for reducing dependence on manual labels and improving representation robustness and cross-scenario generalization [13,14]. Contrastive predictive coding (CPC), proposed by Oord et al. (2018) [15], laid the foundation for self-supervised learning on time-series data. The latest advances in time-series self-supervised learning were reviewed by Zhang et al. (2024) [16]. However, existing studies have mainly focused on specific signal prediction or state classification, and few studies have systematically designed self-supervised learning frameworks for low-noise state representation from the perspective of system risk modeling mechanisms.

The main contributions of this study are summarized as follows:

1. Starting from the essence of system risk modeling, state prediction is transformed from a “label-fitting problem” into a “structural representation learning problem”. A low-noise feature extraction strategy that does not rely on strong manually defined labels is proposed, thereby fundamentally alleviating the problems of high label subjectivity, high construction cost, and severe noise.
2. The characteristics of multi-source heterogeneous sensor information are comprehensively considered. Temperature, humidity, vibration, illumination, current, voltage, and auxiliary environmental indicators are uniformly regarded as sensor signals, thereby strengthening the model’s perception of system microscopic behavior and macroscopic state changes.
3. A unified modeling mechanism consistent with system logic is designed to address key challenges in high-frequency and multimodal sensor data, including asynchronous alignment difficulties, large statistical distribution discrepancies, and complex multi-scale dependencies, thereby enhancing robustness and adaptability in complex environments.
4. Multi-task experiments are conducted to verify the effectiveness, robustness, and interpretability of the proposed method in state prediction, anomaly detection, and system risk ranking tasks, demonstrating strong application potential.

Methodologically, a low-noise feature extraction framework for system state and risk prediction is proposed, which can be summarized as a unified modeling strategy of “multimodal sensing fusion + self-supervised state representation learning”. First, sensor data from different sources are independently embedded and encoded, and asynchronous multi-frequency signals are represented in a unified latent space through a cross-modal temporal alignment mechanism. Subsequently, a temporal masking-based state structure modeling module is constructed, in which key temporal segments are structurally masked to guide the model to learn long-term dependencies and latent uncertainty structures in the system. Furthermore, a state-oriented contrastive learning constraint mechanism is designed, where positive and negative sample pairs are constructed according to system logic to enhance the discriminability of the model across different state stages and risk phases. Finally, a state representation and downstream task alignment strategy is introduced, allowing the pretrained features to better serve practical tasks such as state prediction, anomaly detection, and system risk ranking. The novelty of this study lies in three aspects: first, a self-supervised learning framework is constructed from the perspective of system risk modeling mechanisms rather than mere prediction accuracy; second, multimodal sensing information is organically integrated with state representation learning to improve the completeness of system state perception; and third, temporal masking modeling, state contrastive constraints, and task alignment are jointly employed to obtain lower-noise, more robust, and more interpretable system risk feature representations.

## 2. Related Work

### 2.1. System State Prediction and Anomaly Detection Methods

System state prediction and anomaly detection are key tasks in intelligent monitoring, industrial equipment management, and environmental monitoring. Traditional methods usually rely on statistical features, thresholds, rule-based stratification, or modeled indicators to quantify system risks or states [17]. For example, mean–variance analysis and threshold control methods can be used for preliminary monitoring of sensor signal fluctuations, while decomposing system risk into systematic and component-specific factors helps clarify the sensitivity of different sensors to the overall state [18]. In addition, traditional anomaly detection indicators, such as threshold exceedance or tail anomaly evaluation, usually depend on statistical assumptions or expert-defined settings and assume that system state patterns will remain stable in the future [19]. However, when high-noise, multimodal, multi-frequency, or non-stationary signals are encountered, these methods often exhibit insufficient robustness and struggle to adapt to dynamic changes in complex environments. Deep learning methods have been widely adopted for sensor signal modeling and state prediction. CNNs and LSTMs were shown by Sezer et al. (2020) [20] to outperform traditional machine learning methods in the prediction of complex sensor sequences. LSTM and Transformer models were further applied by Chen et al. (2024) [21] to multimodal sensor data modeling, and the effectiveness of attention mechanisms in capturing long-range dependencies was demonstrated. However, these methods still mainly follow supervised learning paradigms, and their dependence on high-quality labels makes them vulnerable to noise and label inconsistency.

### 2.2. Applications of Deep Learning in Time-Series Sensor Modeling

Deep learning has reshaped time-series sensor modeling. RNN variants such as LSTM can alleviate the vanishing-gradient problem and have become fundamental models for time-series prediction and anomaly detection [22,23]. The effectiveness of LSTM and GRU in long-horizon prediction of sensor data was verified by Bucci (2020) [24]. Transformer models enhance long-range dependency modeling through self-attention mechanisms [25] and have been widely adopted in multi-sensor state prediction tasks. Hybrid models, such as CNN-LSTM and VMD-GRU, have shown stronger performance in modeling multi-scale temporal patterns [26]. In addition, ensemble and stacking methods improve the accuracy and robustness of multi-source sensor anomaly detection by integrating traditional machine learning and deep models [27,28]. Although deep learning performs well in nonlinear modeling, high-dimensional feature processing, and concept drift adaptation, supervised training still depends on large-scale and reliable labels [29]. The need for unlabeled or weakly labeled representation learning in complex system monitoring was further emphasized [30]. The limitations of fixed-window labeling were analyzed from a self-supervised perspective, while the “label horizon paradox” indicated that strict alignment between training labels and inference targets is not necessarily optimal [31].

### 2.3. Multimodal Fusion and Sensor-Based Anomaly Perception

Multimodal fusion and sensor-based anomaly perception provide an important methodological foundation for complex system state modeling. MFGAN proposed by Qu et al. is a representative multimodal anomaly detection framework, in which temporal dependencies and key features within each sensor modality are modeled through an attention-based autoencoder, and adversarial regularization is then introduced to enhance the reconstruction capability for “normal patterns” [32]. Beyond industrial and environmental monitoring, multimodal perception in 3D object recognition and safety monitoring provides transferable structural experience. MSPE-Fusion proposed by Yu et al. performs multi-level perception enhancement and fusion on sensor features such as radar, LiDAR, and cameras, thereby effectively improving detection performance [33]. Although these methods were mainly designed for autonomous driving, their ideas regarding feature alignment, multi-scale fusion, and attention weight allocation can be transferred to multimodal sensor systems, where various signals are regarded as multi-source perception information and integrated through spatiotemporal alignment and attention mechanisms. Multimodal fusion techniques were systematically reviewed by Hangloo and Arora, and fusion strategies were summarized into early fusion, intermediate fusion, and late fusion [34]. Existing studies have shown that, in safety monitoring and anomaly detection tasks, multimodal fusion can enhance system robustness by exploiting redundant information, allowing the system to remain stable even when some modalities are affected by noise or attacks. However, multimodal fusion may also expand the potential attack surface and privacy exposure, especially when sensitive information such as identity, location, or operational behavior is involved.

### 2.4. Exploration of Self-Supervised and Representation Learning in Sensor Data

Self-supervised learning (SSL) has achieved major progress in computer vision and natural language processing by learning representations from unlabeled data. In multimodal sensor applications, SSL has demonstrated potential for denoising, feature robustness, and cross-scenario generalization. Contrastive predictive coding (CPC), proposed by Oord et al. (2018) [15], laid the foundation for self-supervised learning on time-series data. Cross-domain disentangled learning was used to separate shared and specific representations, thereby improving multi-system risk assessment. MF-CLR was proposed by Duan et al. (2024) [35] for multi-frequency sensor data and achieved leading performance in prediction and classification tasks. However, a review of 187 studies by Giantsidi and Tarantola (2025) [36] found that most SSL-based sensor prediction studies focus on single-indicator prediction or directional classification, whereas self-supervised research oriented toward system risk, state uncertainty, and latent anomaly modeling remains insufficient.

## 3. Materials and Method

### 3.1. Data Collection

The dataset constructed in this study is designed for system state prediction and anomaly detection tasks in multimodal sensor systems, with data collected from January 2022 to December 2024. To explicitly define the physical system being monitored, all data were acquired from an Industrial Internet of Things edge computing environment deployed within a large-scale smart manufacturing facility. This specific deployment scenario explains the necessity of integrating diverse sensing modalities, as the operational stability of the edge computing nodes simultaneously depends on internal hardware conditions, robust network routing, stable external physical environments, and continuous human–machine interactions. The data sources include industrial equipment sensors, environmental monitoring sensor networks, edge acquisition devices, and public environmental data interfaces. The dataset covers various specific sensor types. For environmental sensing, we utilized DHT22 temperature (Aosong Electronics Co., Ltd., Guangzhou, China) and humidity sensors alongside BH1750 illumination sensors (ROHM Co., Ltd., Kyoto, Japan) sampled at a base rate of 1 Hz. Device status data were collected using INA219 (Texas Instruments Inc., Dallas, TX, USA) current and voltage sensors and internal hardware monitors sampled at 10 Hz. Network sensing data, including traffic and latency, were captured via edge routers at a 1 Hz rate. Operational behavior data were recorded from industrial touch terminals at variable event-driven rates, while system event logs were extracted asynchronously. These sensors comprehensively reflect changes in system operating states, environmental conditions, and operational behaviors, as shown in Table 1.

Environmental sensing data are used to characterize system operating environments and external interference factors, including temperature, humidity, illumination intensity, and air quality, resulting in a 12-dimensional feature vector. Device status data reflect hardware operating conditions, including power status, processor temperature, and device load level, forming an 18-dimensional feature space. Network sensing data record information transmission stability, including traffic, latency, packet loss rate, and session duration, contributing 10 feature dimensions. Operational and interaction data record operation frequency, input behavior, and response timing, adding 5 dimensions. Event and log data include system logs and sensor alarms, encoded into a 5-dimensional dense vector.

After collection, all data are subjected to timestamp calibration, sensor identifier binding, outlier detection, and missing-value imputation. Regarding data completeness, the overall missing rate across the dataset is approximately 4.2%. However, this varies significantly across different modalities; environmental monitoring sensors exhibit a higher missing rate of approximately 6.5% due to external deployment conditions, whereas system event logs have a negligible missing rate of under 0.5%. This discrepancy highlights the necessity of the robust missing-value imputation strategies employed. Multi-scale temporal windows are then constructed and aligned to a unified base sampling frequency of 1 Hz. This process ensures that environmental sensing data, device status data, network sensing data, operational behavior data, and event logs are aligned within a unified temporal framework, producing a continuous 50-dimensional feature vector per time step. It is crucial to emphasize that the proposed self-supervised framework strictly utilizes unannotated data during the pre-training phase for extracting system state representations, without any access to manual labels. For the evaluation of downstream tasks, such as anomaly detection and state ranking, ground-truth labels were meticulously constructed to minimize individual subjectivity. These labels were generated using a hybrid approach that combined rule-based statistical thresholds, such as the three-sigma rule for continuous sensor signals, with actual retrospective system failure logs to capture definitive fault events. To guarantee the real-world validity of our performance evaluation, we explicitly clarify that all labeled anomalies are based entirely on real physical equipment failures and authentic operational deviations; synthetically injected outliers were strictly excluded from the evaluation dataset. Furthermore, all initial annotations underwent a secondary validation process where experienced maintenance engineers cross-referenced the flagged anomalies with historical maintenance records to ensure the objectivity and accuracy of the ground truth. These validated labels are exclusively reserved for the downstream supervised fine-tuning and testing phases. The dataset exhibits a natural class imbalance typical of real-world industrial environments. Specifically, severe abnormal states constitute approximately 3% of the total records, mild to moderate anomalies account for roughly 8%, and the remaining 89% represent normal operating conditions. Regarding dataset availability, the raw proprietary data cannot be made fully public due to strict industrial confidentiality agreements. However, an anonymized and normalized subset of the dataset, along with the data processing scripts, has been open-sourced to facilitate experimental reproducibility and future research.

### 3.2. Data Augmentation

In multimodal sensor system modeling, data preprocessing and temporal augmentation are key procedures that directly affect the quality of system state representations. Raw sensor sequences contain useful information reflecting system states and potential anomalies, but they are also mixed with missing observations, asynchronous sampling rates, measurement noise, and transmission delays. Proper preprocessing prevents models from incorrectly identifying incidental noise as predictive patterns, thereby ensuring stable performance under extreme events.

Let the original sensor time series be denoted as $X={\left\{x_{t}\right\}}_{t=1}^{T}$, where $x_{t}\in R^{d}$ represents the *d*-dimensional observation vector at time *t*, encompassing temperature, humidity, vibration, voltage, current, device load, and other state indicators. To effectively resolve cross-modal asynchrony, we establish a base temporal alignment window of 1 s, standardizing all multi-source signals to a unified 1 Hz sampling frequency. For high-frequency signals such as device status data sampled at 10 Hz, average pooling downsampling is applied within each 1-s window to extract the local mean. Conversely, for low-frequency or intermittently missing continuous environmental data, linear interpolation based on temporal continuity is utilized to estimate the missing values. The underlying idea is to approximate missing observations by using the local trend of adjacent time points, thereby reducing information loss. If the continuous observation at time *t* is missing and the nearest valid observations exist at $t_{1}<t<t_{2}$, the interpolation can be expressed as

(1) $${\hat{x}}_{t}=x_{t_{1}}+\frac{t-t_{1}}{t_{2}-t_{1}}\left(x_{t_{2}}-x_{t_{1}}\right).$$

For discrete signals such as event logs and operational behavior data, a forward-fill policy is adopted to maintain the last known valid state until a new event occurs. For more complex missing cases in continuous signals, a sliding-window local mean can be used for robust imputation. Let the window radius be *k*; then the estimate of the missing position *t* can be written as

(2) $${\hat{x}}_{t}=\frac{1}{|\Omega_{t}|}\sum_{i\in \Omega_{t}}x_{i},\;\Omega_{t}=\left\{i|t-k\leq i\leq t+k,\;x_{i}\;\mathrm{is}\;\mathrm{observable}\right\}.$$

Furthermore, in practical scenarios where an entire sensor modality is temporarily delayed or completely missing, we address this by zero-padding the input features of that specific modality and simultaneously concatenating a binary missingness mask vector to the input. This mask indicator explicitly informs the downstream temporal and cross-modal attention mechanisms to ignore the zero-padded values, thereby guaranteeing that the model computation remains uninterrupted and stable.

To eliminate the influence of amplitude differences among different sensor signals, standardization or scale transformation is usually required for each dimension. For abnormal fluctuations or heavy-tailed phenomena, robust standardization can be adopted, where the median and interquartile range are used instead of the mean and standard deviation. The calculation is given as follows:

(3) $${\tilde{x}}_{t}=\frac{x_{t}-\mathrm{Med}\left(x\right)}{Q_{3}\left(x\right)-Q_{1}\left(x\right)+\epsilon },$$

where Med(x) denotes the median, $Q_{1}\left(x\right)$ and $Q_{3}\left(x\right)$ denote the first and third quartiles, respectively, and ϵ is a small constant used to prevent division by zero. For relatively stationary signals, rolling mean and rolling standard deviation can also be used for dynamic standardization:

(4) $${\tilde{x}}_{t}=\frac{x_{t}-\mu_{t}^{\left(w\right)}}{\sigma_{t}^{\left(w\right)}+\epsilon },\;\mu_{t}^{\left(w\right)}=\frac{1}{w}\sum_{i=t-w+1}^{t}x_{i},\;\sigma_{t}^{\left(w\right)}=\sqrt{\frac{1}{w}\sum_{i=t-w+1}^{t}{\left(x_{i}-\mu_{t}^{\left(w\right)}\right)}^{2}}.$$

Outlier processing is also a critical step. Extreme observations can be mapped into a bounded interval through quantile clipping or hyperbolic tangent compression, thereby reducing numerical instability during training:

(5) $$x_{t}^{\mathrm{clip}}=\left\{\begin{array}{cc} q_{\alpha }, & x_{t}<q_{\alpha },\\ x_{t}, & q_{\alpha }\leq x_{t}\leq q_{1-\alpha },\\ q_{1-\alpha }, & x_{t}>q_{1-\alpha }, \end{array}\right.$$

or

(6) $$x_{t}^{\prime }=\tanh\left(\beta x_{t}\right),$$

where β denotes the compression intensity.

After basic preprocessing, temporal augmentation is used to generate structurally consistent multi-view samples for self-supervised or contrastive learning. Given an observation window at time step *t* denoted as $X_{t}={\left\{x_{i}\right\}}_{i=t}^{t+L-1}$ with a total window length *L*, the augmentation methods include temporal window cropping, where continuous subsequences of length *l* are extracted from the original sequence so that the model can learn state consistency under different observation windows:

(7) $$A_{\mathrm{crop}}\left(X_{t}\right)=X_{s:s+l-1},\;t\leq s\leq t+L-l$$

Amplitude scaling is used to simulate amplitude variations under different environmental conditions, where a scaling tolerance parameter δ defines the maximum amplitude variation bound:

(8) $$A_{\mathrm{scale}}\left(x_{t}\right)=\lambda x_{t},\;\lambda \in \left[1-\delta ,1+\delta \right]$$

Local temporal shifting is used to simulate slight delays in sensor signal arrival, where the maximum permissible temporal shift step is constrained by the parameter τ:

(9) $$A_{\mathrm{shift}}\left(x_{t}\right)=x_{t+\Delta_{t}},\;\Delta_{t}\in \left[-\tau ,\tau \right]$$

State-similar segment replacement is further used, in which segments with similar system states are extracted from different sequences and concatenated:

(10) $$A_{\mathrm{replace}}\left(X^{\left(a\right)}\right)=\left[x_{t_{1}:t_{2}}^{\left(a\right)},x_{t_{2}+1:t_{3}}^{\left(b\right)},x_{t_{3}+1:t_{4}}^{\left(a\right)}\right]$$

In self-supervised or contrastive learning scenarios, two augmented views can be generated from the same sequence, denoted as $X^{\left(1\right)}=A_{1}\left(X\right)$ and $X^{\left(2\right)}=A_{2}\left(X\right)$. The encoder outputs $z^{\left(1\right)}$ and $z^{\left(2\right)}$, and their consistency is constrained by cosine similarity:

(11) $$\mathrm{sim}\left(z^{\left(1\right)},z^{\left(2\right)}\right)=\frac{z^{(1)⊤}z^{\left(2\right)}}{|z^{\left(1\right)}\left|\right|z^{\left(2\right)}|}$$

Overall, two principles are followed in the data preprocessing and temporal augmentation strategy. First, noise, drift, and scale differences are reduced as much as possible while preserving the dynamic characteristics, relative variation relationships, and system state structures of sensor time series. Second, augmentation operations emphasize structural consistency rather than random perturbation. Diverse training samples are generated through cropping, scaling, temporal shifting, and state-similar segment replacement, thereby improving the ability of the model to learn robust and low-noise system state representations in complex environments.

### 3.3. Proposed Method

#### 3.3.1. Overall

The proposed method is constructed as a unified framework consisting of “multimodal sensor signal encoding → temporal masking-based self-supervised modeling → state-contrastive representation constraint → downstream task-aligned prediction”. Given multi-source sensor samples that have been temporally aligned and feature-normalized, signals from different modalities, including environmental sensing data, device status data, operational behavior data, network transmission data, and event log data, are first fed into their corresponding modality encoders. For continuous temporal modalities, temporal convolution and attention-based encoding structures are adopted to extract local fluctuation patterns and long-range dependencies. For event logs or textual description modalities, semantic encoders are used to extract event impacts, alarm information, and clues related to system state changes. Subsequently, modality-specific features are mapped into a unified latent space, and the importance weights of different modalities under the current system state are calculated through a cross-modal attention fusion module, thereby forming a comprehensive system state representation. This representation is not directly fed into a supervised predictor, but is first introduced into the self-supervised state representation learning stage. In this stage, several key temporal segments in the input sequence are masked, forcing the encoder to recover the masked content based on unmasked environmental changes, device states, operational behaviors, network transmission signals, and event log signals. In this way, intrinsic dependencies and latent state structures among different temporal segments can be learned. The contextual representation output by the temporal masking module is further fed into the state-oriented contrastive learning module. Positive and negative sample pairs are constructed according to local fluctuation intensity, device load abnormality, operational behavior changes, network transmission states, and event-stage similarity, so that samples under similar system states are pulled closer in the representation space, whereas samples under different states are effectively separated. Consequently, the interference of short-term noise and incidental fluctuations in representation learning can be reduced. After self-supervised pretraining is completed, the learned low-noise state representation is transferred to the downstream task alignment module. Corresponding prediction heads are designed for different system state perception tasks, including system state regression, abnormal event classification, multi-source anomaly scoring, and state risk ranking, so that the general state representation can be matched with specific monitoring, early warning, and operation-and-maintenance decision objectives. During the overall training process, the masked reconstruction objective is used to constrain the model to learn temporal structures, the contrastive learning objective is used to optimize the state representation space, and the downstream task objective is used to calibrate predictive value. These three objectives jointly enable stable, transferable, and interpretable system state features to be extracted from multimodal sensor signals.

#### 3.3.2. Temporal Masking-Based State Structure Modeling Module

The temporal masking-based state structure modeling module follows a self-supervised learning process of “masked input → encoded representation → decoded reconstruction → structural constraint”. Its core objective is to guide the model to learn stable long-term system state structures from multimodal sensor sequences without relying on manually defined state labels. Let the aligned input sequence be denoted as $X={\left\{x_{t}\right\}}_{t=1}^{T}$, where $x_{t}\in R^{d}$ represents the multimodal system state vector at time *t*, containing environmental, device, operational, network, and event-related features. First, a binary mask vector $m_{t}\in \left\{0,1\right\}$ is generated according to the masking strategy, and the masked input is obtained as ${\tilde{x}}_{t}=m_{t}x_{t}+\left(1-m_{t}\right)e_{\mathrm{mask}}$, where $e_{\mathrm{mask}}$ denotes a learnable mask embedding. Unlike ordinary random masking, the masking units in this study mainly consist of continuous temporal segments, and state-related indicators such as local state fluctuation intensity, device load abnormality, network latency variation, and operational behavior abnormality are incorporated to adjust the masking probability, making high-uncertainty regions more likely to be masked. This design forces the model to recover missing information according to the system states before and after the masked segments, cross-modal collaborative relationships, and long-term temporal dependencies, thereby preventing the model from merely memorizing local noise or instantaneous anomalies.

**Figure 1:**
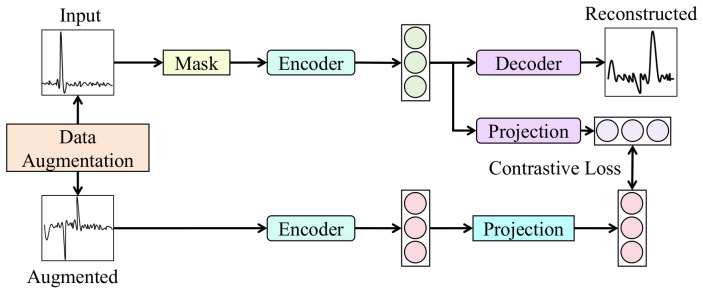
The temporal masking-based state structure modeling module learns stable low-noise system state representations through temporal masking, encoded reconstruction, and contrastive constraints on augmented views.

As shown in Figure 1, from the perspective of network structure, the masked sequence is first fed into the temporal encoder $f_{\theta }\left(\cdot \right)$ to obtain the contextual latent representation $H=f_{\theta }\left(\tilde{X}\right)={\left\{h_{t}\right\}}_{t=1}^{T}$. The encoder consists of a modality embedding layer, a positional encoding layer, and multiple temporal attention blocks. The modality embedding layer projects sensor signals from different sources into a unified dimension $d_{h}$, the positional encoding layer is used to preserve the temporal order of sensor sequences, and the temporal attention blocks calculate dependencies among different temporal segments through query, key, and value mappings. The basic form is given by $\mathrm{Attention}\left(Q,K,V\right)=\mathrm{softmax}\left(QK^{⊤}/\sqrt{d_{k}}\right)V$. Through this structure, associations between masked and unmasked segments can be established over the global temporal range, while short-term state fluctuations and long-term anomaly accumulation processes can be captured simultaneously. Subsequently, the latent representation *H* is fed into the decoder $g_{\phi }\left(\cdot \right)$ to reconstruct the original observations at the masked positions, yielding ${\hat{x}}_{t}=g_{\phi }\left(h_{t}\right)$. A lightweight multilayer perceptron or reverse temporal attention structure is adopted as the decoder to avoid directly copying input noise due to excessive model capacity. The reconstruction loss is calculated only at the masked positions and can be expressed as $L_{rec}=\frac{1}{\left|M\right|}\sum_{t\in M}{∥x_{t}-{\hat{x}}_{t}∥}_{2}^{2}$, where M denotes the set of masked temporal indices. For state-sensitive variables such as temperature, vibration, current, load, and latency, a weighted reconstruction form can also be introduced as $L_{wrec}=\frac{1}{\left|M\right|}\sum_{t\in M}\omega_{t}{∥x_{t}-{\hat{x}}_{t}∥}_{2}^{2}$, where $\omega_{t}$ is determined by local abnormality intensity, enabling the model to focus more on temporal segments with significant system state changes.

From a mathematical perspective, temporal masking modeling is essentially intended to maximize the conditional dependence between masked segments and contextual segments, namely to learn $p_{\theta }\left(x_{M}|x_{\bar{M}}\right)$. In high-noise sensor environments, random micro-level perturbations are often difficult to predict stably from context, whereas state information jointly determined by system structure, device operating status, environmental changes, and event triggers possesses stronger conditional recoverability. Therefore, when the model is required to recover masked segments from context, structurally consistent state factors are more likely to be preserved, while the influence of unpredictable noise is weakened. This module is also connected to the subsequent contrastive learning branch. The latent representation output by the encoder is fed into the reconstruction decoder to calculate the reconstruction constraint on the one hand, and is used as the state representation input to the projection layer for consistency constraints with augmented-view representations on the other hand. Therefore, the model can learn not only “how to recover the masked system state”, but also “which structures remain stable under different perturbations”. This design is particularly suitable for the system state prediction and anomaly detection tasks in this study, because abnormal states are usually not single-point noise, but the result of gradual accumulation and coupled changes in multimodal signals over time. Through masked reconstruction, dynamic associations among environmental disturbances, device loads, network transmission, operational behaviors, and event logs can be explicitly modeled, thereby yielding low-noise, transferable, and more interpretable state structure representations.

#### 3.3.3. State-Oriented Contrastive Learning Representation Constraint Mechanism

The state-oriented contrastive learning representation constraint mechanism adopts a dual-branch encoding projection similarity constraint structure. However, its design focus is not arbitrary augmentation of original samples, but the construction of multi-view representations with state consistency around system state semantics.

**Figure 2:**
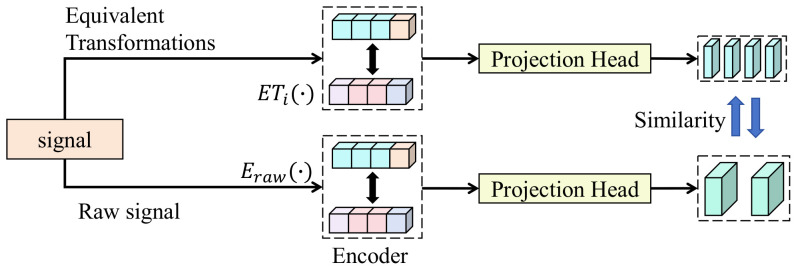
The state-oriented contrastive learning representation constraint mechanism generates multi-view representations through state-consistent transformations and employs contrastive learning to enhance discrimination and stability across different system states.

As shown in Figure 2, let the input multimodal sensor state sequence be denoted as $X\in R^{T\times C}$, where *T* represents the temporal length and *C* represents the number of fused channels. First, a set of state-consistent transformations $T=\{T_{ta},T_{ap},T_{fft},T_{emd}\}$ is constructed for the original sequence, corresponding to local temporal perturbation, amplitude-preserving scaling, frequency-domain low-noise enhancement, and empirical mode decomposition reconstruction, respectively. After transformation, multiple state-consistent views $\left\{X_{ta},X_{ap},X_{fft},X_{emd}\right\}$ are obtained, while the original view $X_{raw}$ is retained. These views differ in short-term noise, local amplitude, or frequency-domain details, but their latent system states should remain consistent; thus, they are defined as positive sample relationships. Each augmented view is fed into the shared-parameter state encoder $E_{\theta }\left(\cdot \right)$, while the original view is fed into the baseline encoder $E_{\xi }\left(\cdot \right)$, which has an independent but structurally identical architecture. The encoder consists of $L_{e}$ one-dimensional temporal convolution blocks, $L_{a}$ multi-head temporal attention blocks, and a global state pooling layer. The input size of the *l*-th convolution block is $T_{l}\times C_{l}$, the kernel size is $k_{l}$, the stride is $s_{l}$, and the output channel number is $C_{l+1}$. Therefore, the output width can be expressed as $T_{l+1}=\left\lfloor \left(T_{l}+2p_{l}-k_{l}\right)/s_{l}\right\rfloor +1$, where $p_{l}$ denotes the padding size applied to the convolution block to preserve temporal boundaries, and the output feature is denoted as $H_{l}\in R^{T_{l+1}\times C_{l+1}}$. Subsequently, the attention layer maps state dependencies along the temporal dimension into the latent dimension *D*, and the output representation is denoted as $h\in R^{D}$. To prevent contrastive learning from directly constraining backbone features and impairing downstream predictive capability, the encoded result is further fed into the projection head $P_{\psi }\left(\cdot \right)$. The projection head consists of $L_{p}$ fully connected layers, with the width of the *j*-th layer denoted as $d_{j}$, and the final output is $z\in R^{d_{z}}$. This design allows the backbone encoder to retain interpretable state factors, while the projection space mainly undertakes contrastive optimization.

For any original sample $X_{i}$, its original representation and augmented representation are respectively defined as

(12) $$z_{i}^{raw}=P_{\psi }\left(E_{\xi }\left(X_{i}\right)\right),\;z_{i}^{m}=P_{\psi }\left(E_{\theta }\left(T_{m}\left(X_{i}\right)\right)\right),\;T_{m}\in T.$$

To make the similarity constraint more consistent with system state logic, an ordinary instance-level contrastive loss is not directly adopted. Instead, state similarity weights are introduced. Let the state statistics of samples *i* and *j* be denoted as $s_{i}$ and $s_{j}$, respectively. It is imperative to clarify that these state statistics are purely localized, unsupervised mathematical features calculated dynamically from the raw input signals within the current temporal window, such as local signal variance, peak-to-peak amplitude differences, and gradient change rates. We explicitly declare that the computation of these statistical weights has absolutely no access to any ground-truth anomaly labels utilized in downstream tasks, nor does it rely on any predefined anomaly thresholds or prior expert definitions. By solely utilizing the intrinsic physical data distributions to guide the sample weighting, this design fundamentally eliminates any possibility of label leakage, thereby guaranteeing the absolute fairness and strictly self-supervised nature of the representation learning process. The state-consistency weight between them is then defined as

(13) $$\omega_{ij}=\exp\left(-\frac{∥s_{i}-s_{j}{∥}_{2}^{2}}{\gamma }\right),$$

where γ is the smoothing parameter for state similarity. When the system states of two samples are closer, $\omega_{ij}$ becomes larger, and the model tends to pull them closer in the representation space. Furthermore, the state-oriented contrastive objective between the augmented views and the original view can be formulated as

(14) $$L_{state}=-\frac{1}{N}\sum_{i=1}^{N}\log\frac{\sum_{m\in T}\exp\left(\omega_{ii}\cdot \kappa \left(z_{i}^{raw},z_{i}^{m}\right)/\tau \right)}{\sum_{j=1}^{N}\sum_{m\in T}\exp\left(\omega_{ij}\cdot \kappa \left(z_{i}^{raw},z_{j}^{m}\right)/\tau \right)},$$

where κ(·,·) denotes normalized inner-product similarity and τ denotes the temperature coefficient. Unlike ordinary contrastive learning, this loss does not simply distinguish sample identities, but adjusts positive and negative sample boundaries through state similarity, enabling the model to focus more on whether the system state structures are consistent rather than whether the samples originate from the same temporal window.

The mathematical rationality of this design can be explained from the perspective of representation invariance. If the state-consistent transformation $T_{m}$ does not change the latent state variable $y_{i}$, namely $p\left(y_{i}|X_{i}\right)=p\left(y_{i}|T_{m}\left(X_{i}\right)\right)$, the ideal encoder should satisfy

(15) $$E_{\theta }\left(X_{i}\right)=E_{\theta }\left(T_{m}\left(X_{i}\right)\right)+\epsilon_{m},\;E\left[\epsilon_{m}\right]=0.$$

When $L_{state}$ is minimized, the positive sample term increases $\kappa (z_{i}^{raw},z_{i}^{m})$, and the following constraint tendency can therefore be obtained:

(16) $$\arg\min_{\theta ,\psi }L_{state}\Rightarrow E_{m\in T}\left[∥z_{i}^{raw}-z_{i}^{m}{∥}_{2}^{2}\right]\to 0.$$

Meanwhile, for samples with large state differences, a larger $∥s_{i}-s_{j}{∥}_{2}$ forms a stronger separation boundary in the denominator, encouraging the representations to satisfy

(17) $${∥z_{i}-z_{j}∥}_{2}^{2}\geq \Delta \left(s_{i},s_{j}\right),$$

where $\Delta (s_{i},s_{j})$ is a margin function that increases with state differences. Accordingly, this module actually constructs a state-aware metric structure in the representation space. Multiple perturbed views under the same state semantics are compressed into adjacent regions, while samples from different anomaly stages or operating states are pushed farther apart. When applied to the task in this study, this mechanism can effectively reduce the interference of random noise, local spikes, and asynchronous sampling errors in multimodal sensor data during representation learning. The model no longer relies on local changes from a single sensor, but extracts state factors from multi-source sensing signals that remain stable across temporal windows, devices, and environments. Compared with a self-supervised objective that relies only on masked reconstruction, the state-oriented contrastive constraint further enhances the discriminability of the representation space, allowing subsequent system state prediction, abnormal event classification, and state risk ranking tasks to obtain clearer, lower-noise, and physically interpretable input features.

#### 3.3.4. State Representation and Downstream Prediction Task Alignment Strategy

The state representation and downstream prediction task alignment strategy adopts a hierarchical design of “shared state encoding–learnable mapping–mixture-of-experts prediction” in its overall structure, enabling the general state representation obtained in the self-supervised stage to be effectively transformed into task-specific features required for specific monitoring, early warning, and operation-and-maintenance tasks.

**Figure 3:**
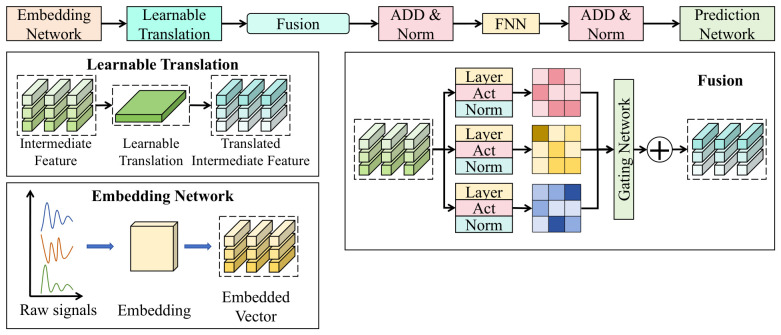
The state representation and downstream prediction task alignment strategy aligns learned system state representations with multi-task prediction objectives through learnable transformations and a mixture-of-experts mechanism for adaptive anomaly detection and dynamic system state prediction.

As shown in Figure 3, let the unified state representation obtained from the preceding modules be denoted as $Z\in R^{T\times D}$. It is first fed into the embedding network $E_{\phi }\left(\cdot \right)$ for feature compression and reconstruction, yielding the basic embedding vector $H\in R^{T\times d_{h}}$. The embedding network consists of multiple linear transformations and nonlinear activations, and the width of each layer is denoted as $d_{l}$. Through layer-wise projection, high-dimensional state factors are mapped into a compact task space. Subsequently, a learnable mapping module $T_{\psi }\left(\cdot \right)$ is introduced to structurally transform the intermediate features, which can be expressed as

(18) $$\tilde{H}=T_{\psi }\left(H\right)=\sigma \left(HW_{t}+b_{t}\right),$$

where $W_{t}$ denotes the transformation matrix and σ(·) denotes the nonlinear activation function. This module is used to eliminate the distributional shift between self-supervised representations and downstream tasks, making the state factors semantically closer to the prediction targets.

In the prediction stage, a state prediction network based on a mixture-of-experts mechanism is adopted to improve the adaptability of the model under different system operating conditions. Let several sub-prediction experts be denoted as $f_{k}\left(\cdot \right)$. Each expert is used to model a specific state pattern, such as a normal operating phase, high-load phase, network disturbance phase, device anomaly phase, or event-driven phase. Its output is given by

(19) $$y_{k}=f_{k}\left(\tilde{H}\right),$$

where $f_{k}\left(\cdot \right)$ is composed of a multilayer feedforward network, and the hidden dimension of each layer is denoted as $d_{f}$. Complex system state patterns can therefore be captured through nonlinear mapping. To dynamically select the most appropriate expert, a gating network $G_{\omega }\left(\cdot \right)$ is constructed to generate expert weights according to the current state representation:

(20) $$p_{k}=\frac{\exp\left(g_{k}\left(\tilde{H}\right)\right)}{\sum_{j}\exp\left(g_{j}\left(\tilde{H}\right)\right)},$$

where $g_{k}\left(\cdot \right)$ denotes the gating function. The final prediction is obtained as a weighted combination of the outputs of all experts:

(21) $$\hat{y}=\sum_{k}p_{k}y_{k}.$$

From a mathematical perspective, this structure is equivalent to decomposing the conditional distribution p(y|Z) as

(22) $$p\left(y|Z\right)=\sum_{k}p_{k}\left(Z\right)\;p\left(y|Z,k\right),$$

where $p_{k}\left(Z\right)$ is produced by the gating network, and p(y|Z,k) is modeled by the corresponding expert. This decomposition can effectively characterize the property that different operating states in sensor systems correspond to different generation mechanisms. When the system is in different phases, the gating network automatically adjusts the expert weights, enabling the model to select the most appropriate state pattern for prediction and thus avoiding the limitation that a single model is difficult to adapt to changing environments.

To achieve effective alignment between representations and tasks, a joint optimization objective is introduced during training, so that the state representation not only satisfies self-supervised structural constraints but also minimizes the downstream task loss $L_{task}$. The overall optimization objective can be written as

(23) $$L=L_{self}+\lambda L_{task},$$

where $L_{self}$ denotes the self-supervised loss from the preceding modules, and λ is the trade-off coefficient. This objective ensures that the encoder gradually adjusts the representation space to downstream prediction tasks while preserving general structural information. To explicitly address the severe class imbalance problem inherent in real-world anomaly detection, where normal operating data vastly outnumbers anomalous events, a specialized design is adopted for the downstream task loss $L_{task}$. Rather than using a standard cross-entropy loss, $L_{task}$ is implemented as a Focal Loss. This cost-sensitive learning objective dynamically scales the gradients based on prediction confidence, heavily penalizing missed detections of the rare but critical anomaly class while down-weighting the easily classified normal samples. Furthermore, the inherent mechanism of the preceding self-supervised pretraining naturally mitigates the imbalance issue. By predominantly learning robust normal state representations from the abundant normal data, the model establishes a well-defined normal feature manifold. Consequently, during the fine-tuning phase, even a minuscule fraction of anomalous samples can form distinct clusters distinctly separated from the normal manifold, significantly improving the sensitivity of the downstream alignment strategy to rare events.

To explicitly quantify the system risk during the inference phase, the final anomaly score used to trigger an alarm is mathematically defined as a weighted combination of the downstream prediction output and the self-supervised reconstruction error. Let $AS_{t}$ denote the anomaly score at time step *t*. It is calculated as

(24) $$AS_{t}=\eta {\hat{y}}_{t}+\left(1-\eta \right)E_{rec,t},$$

where ${\hat{y}}_{t}$ represents the anomaly risk probability output by the mixture-of-experts prediction network, $E_{rec,t}$ denotes the normalized reconstruction error from the temporal masking module at time *t*, and η is a balancing hyperparameter. Furthermore, the alarm threshold for this anomaly score is not manually preset. Instead, it is dynamically determined using the Peak-over-Threshold method on the validation set. This approach applies Extreme Value Theory to fit the tail distribution of the anomaly scores and automatically selects the optimal threshold that maximizes the F1-score, thereby ensuring that the alarm triggering mechanism remains objective, data-driven, and highly adaptable to varying system noise levels.

This design offers three advantages for the task in this study. First, through the embedding and learnable mapping modules, self-supervised state representations are effectively transformed into a feature space with task semantics, thereby avoiding misalignment between representations and prediction targets. Second, the mixture-of-experts structure can characterize the diversity of system state changes, allowing the model to automatically select the optimal prediction path under different operating conditions and thus improving robustness and generalization capability. Finally, the gating mechanism enables dynamic adaptability. When facing high-noise sensor data, structural breaks, and cross-scenario transfer, the model can adjust its decision logic according to the current system state, thereby achieving more stable and accurate system state prediction and anomaly detection.

## 4. Results and Discussion

### 4.1. Experimental Configuration

#### 4.1.1. Hardware and Software Platform

The experiments were conducted on a high-performance deep learning workstation. On the server side, an Intel Xeon Silver multi-core CPU with a clock frequency of no less than 2.4GHz was used to support large-scale multimodal sensor time-series data loading, preprocessing, and parallel training. The graphics processing unit was an NVIDIA RTX 4090 or an equivalent GPU with 24GB of video memory, which was sufficient for batch training and attention computation of multimodal sensor sequences under the Transformer framework. The system memory was configured as 128GB to ensure efficient caching and rapid access to multi-source data, including environmental sensing data, device status data, operational behavior data, network transmission data, and event logs during joint modeling. A 2TB NVMe SSD was used for local storage to improve the read and write efficiency of large-scale sensor data slicing, model parameter saving, and experimental log recording. Overall, the hardware platform provided sufficient support for training and validating the proposed model under multimodal and multi-frequency sensor system settings, while ensuring experimental stability and reproducibility.

In terms of the software platform, the experiments were implemented on Ubuntu 22.04 LTS. PyTorch 2.2 was adopted as the deep learning framework, and CUDA 12.1 and cuDNN acceleration libraries were used during training to improve the efficiency of tensor operations and attention computation. Python 3.10 was used as the programming language. NumPy, Pandas, and SciPy were mainly used for sensor sequence cleaning, sliding-window construction, and statistical feature calculation. Scikit-learn was used for metric calculation and experimental analysis, while Matplotlib 3.10.8 and Seaborn 0.13.2 were adopted for model visualization and result plotting. To ensure consistency of experimental results, random seeds were fixed during training, and pseudo-random number generation states, deterministic GPU operation options, and logging modules were uniformly configured in the software environment, thereby reducing result fluctuations caused by differences in runtime environments. Although these measures successfully eliminated macroscopic variances, we acknowledge that minuscule remaining fluctuations could not be completely eradicated. We have identified that these residual variations are entirely comprehensible and originate from the inherent nondeterminism of atomic operations at the hardware level during the parallel computation of attention matrices on GPUs, alongside microscopic timing differences in multi-threaded asynchronous data loading. However, these remaining fluctuations are strictly confined to an insignificant numerical scale and do not affect the overall reliability, reproducibility, or validity of our experimental conclusions.

For dataset partitioning and hyperparameter settings, sensor samples were divided in chronological order to avoid future information leakage. To address the inherent class imbalance, a temporally constrained stratified sampling strategy was adopted during the dataset partitioning. The overall dataset was split into training, validation, and test sets at a ratio of 7:1:2, ensuring that the approximate proportions of severe anomalies, mild anomalies, and normal states were consistently maintained across all subsets, particularly within the validation and test sets, without violating the temporal causality of the sequences. The training set was used for model parameter learning, the validation set was used for model selection and early stopping, and the test set was used for final performance evaluation. Considering the phased characteristics and distribution drift of sensor data, a 5-fold cross-validation strategy was further adopted during training. Specifically, 5 rolling subsets were constructed within the training samples in chronological order, and model training and validation were alternately performed to evaluate the generalization capability of the model under different operating states more robustly. The input temporal window length was set to 60, indicating that the model used historical information from 60 consecutive time steps to predict future system states. The embedding dimension was set to 128, the number of multi-head attention heads was set to 4, the number of Transformer encoder layers was set to 3, and the hidden dimension of the feed-forward network was set to 256. AdamW was adopted as the optimizer, with an initial learning rate of 0.0001, a weight decay coefficient of 0.00001, a batch size of 32, and a maximum of 100 training epochs. Early stopping was triggered when validation performance did not improve for 10 consecutive epochs. To enhance training stability, the dropout ratio was set to 0.1, and the gradient clipping threshold was set to 1.0. In the self-supervised pretraining stage, the temporal masking ratio was set to 0.15, the contrastive learning temperature parameter was set to 0.07, and the weighting coefficient for the state representation and downstream task alignment loss was set to 0.3. Rather than being empirically assigned, these hyperparameter values were systematically optimized through a grid search strategy evaluated on the validation set. To provide practical tuning guidance and best practices for comparable high-noise sensor environments, we detail the optimization rationale. The temporal masking ratio was searched across a range from 0.05 to 0.30. The optimal value of 0.15 was selected because it perfectly balances the retention of contextual information against the difficulty of the reconstruction task, preventing the model from merely memorizing local noise while forcing it to learn stable global structures. Furthermore, the contrastive temperature parameter was evaluated between 0.01 and 0.15. A value of 0.07 was systematically chosen as it provides sufficient sharpness in the representation space to distinguish between normal fluctuations and genuine anomaly signatures under strong noise interference. The architectural parameters, including the number of attention heads and network layers, were derived by evaluating the trade-off between anomaly detection accuracy and the computational latency constraints required for practical edge deployment. Consequently, these parameter settings reproducibly address the strong noise, local fluctuations, and computational complexity of multimodal sensor data fusion, thereby ensuring model expressiveness while controlling training costs and improving adaptability in real system state prediction tasks.

#### 4.1.2. Baseline Models and Evaluation Metrics

The baseline models included GARCH [37], LSTM [38], MLP [39], Transformer [25], TCN [40], CPC [41], TNC [42], TS2Vec [43], and MF-CLR [35]. GARCH characterizes the volatility clustering of time-series signals through recursive conditional variance modeling and provides strong interpretability. LSTM can preserve historical information over relatively long temporal ranges in sequence modeling and is suitable for capturing complex temporal dependencies. MLP, as a feed-forward fully connected neural network, takes flattened multidimensional features within a sliding window as input and is simple in structure and stable during training, making it suitable as a basic comparison model. Transformer is based on the self-attention mechanism and can directly model global dependencies among different temporal positions, making it suitable for high-dimensional multivariate time-series data. TCN uses causal convolution and dilated convolution to expand the receptive field while maintaining temporal causality, thereby effectively modeling dynamic patterns at different temporal scales with high parallel computational efficiency. To further validate the advantages of the proposed framework in self-supervised representation learning, state-of-the-art self-supervised time-series models were introduced. CPC extracts useful representations from high-dimensional data by predicting future observations in latent space using autoregressive models. TNC learns representations by ensuring that the distribution of signals within a temporal neighborhood is distinguishable from non-neighboring signals. TS2Vec is a universal framework that learns robust contextual representations for time series through hierarchical contrastive learning over augmented context views. MF-CLR enhances time-series representation by integrating frequency-domain information and designing cross-frequency contrastive objectives.

The evaluation metrics included mean squared error MSE, mean absolute error MAE, root mean square error RMSE, information coefficient IC, rank information coefficient RankIC, the classification discrimination metric AUC, and the standard anomaly detection metric F1. MSE, MAE, and RMSE were used to measure the prediction accuracy of continuous system states or anomaly intensities. In complex industrial sensor systems, identifying binary anomalies is often insufficient. It is equally critical to accurately rank the risk severity across different devices or temporal windows to determine maintenance priorities and resource allocation. Therefore, IC and RankIC were chosen to characterize the monotonic correlation between the predicted risk scores and the actual anomaly severity degrees, effectively evaluating the ability of the model to rank system state magnitudes. To comprehensively assess deterministic binary anomaly classification performance commonly expected in structural health monitoring, F1 and AUC were utilized. The metrics are defined as follows:

(25) $$MSE=\frac{1}{N}\sum_{i=1}^{N}{\left({\hat{y}}_{i}-y_{i}\right)}^{2}$$

(26) $$MAE=\frac{1}{N}\sum_{i=1}^{N}\left|{\hat{y}}_{i}-y_{i}\right|$$

(27) $$RMSE=\sqrt{\frac{1}{N}\sum_{i=1}^{N}{\left({\hat{y}}_{i}-y_{i}\right)}^{2}}$$

(28) $$IC=\frac{\sum_{i=1}^{N}\left({\hat{y}}_{i}-\bar{\hat{y}}\right)\left(y_{i}-\bar{y}\right)}{\sqrt{\sum_{i=1}^{N}{\left({\hat{y}}_{i}-\bar{\hat{y}}\right)}^{2}}\sqrt{\sum_{i=1}^{N}{\left(y_{i}-\bar{y}\right)}^{2}}}$$

(29) $$RankIC=1-\frac{6\sum_{i=1}^{N}d_{i}^{2}}{N(N^{2}-1)}$$

(30) $$AUC=\int_{0}^{1}TPR\left(FPR^{-1}\left(u\right)\right)\;du$$

(31) $$F1=\frac{2\times Precision\times Recall}{Precision+Recall}$$

where *N* denotes the number of samples, $y_{i}$ denotes the true system state or anomaly value of the *i*-th sample, ${\hat{y}}_{i}$ denotes the model prediction, $\bar{y}$ and $\bar{\hat{y}}$ denote the sample means of the true and predicted values, respectively, $d_{i}$ denotes the rank difference between the true ranking and the predicted ranking of the *i*-th sample, TPR denotes the true positive rate which is also referred to as Recall, FPR denotes the false positive rate, Precision denotes the positive predictive value, and *u* is the integration variable. When a discrete approximation is used, AUC can be interpreted as the area under the ROC curve.

### 4.2. Performance Comparison of Different Models

This experiment was designed to verify the effectiveness of the proposed method in system state prediction and anomaly detection from three perspectives: overall prediction accuracy, state-ranking capability, and discrimination between high-abnormality and low-abnormality states.

**Figure 4:**
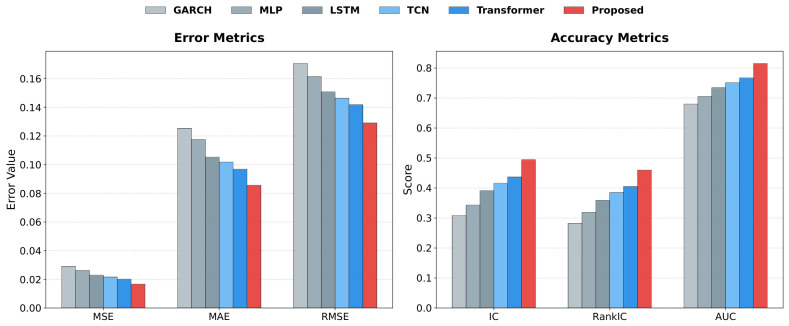
Comparison of error and accuracy metrics among different models for multimodal sensor-based system state prediction, where the proposed method achieves the best performance in both error reduction and state discrimination.

As shown in Table 2 and Figure 4, GARCH achieved MSE, MAE, and RMSE values of 0.0291, 0.1254, and 0.1705, respectively, while its IC, RankIC, and F1 values were 0.308, 0.282, and 0.582, respectively, indicating the weakest overall performance. This result suggests that traditional statistical methods can characterize local fluctuations to some extent, but are difficult to adapt to multimodal, high-noise, and strongly nonlinear sensor data scenarios. Compared with GARCH, MLP achieved a certain improvement, indicating that nonlinear mapping can enhance the fitting capability for state features. However, due to the lack of explicit temporal structure modeling, long-range dynamic dependencies remain difficult to capture. The errors of LSTM were further reduced, showing that the gated recurrent structure can preserve historical information and effectively model dynamic dependencies. TCN performed slightly better than LSTM, suggesting that causal convolution and dilated convolution can enlarge the receptive field while preserving temporal order. Transformer achieved the best performance among the supervised baseline models, with MSE reduced to 0.0201 and F1 reaching 0.695, demonstrating the advantages of self-attention mechanisms in capturing multivariate interactions. Furthermore, the newly introduced self-supervised baselines demonstrated superior performance compared to traditional supervised models. CPC and TNC achieved lower prediction errors and higher state discrimination than Transformer, proving that self-supervised objectives such as predictive coding and neighborhood contrast can learn more robust representations. TS2Vec and MF-CLR further improved the results, with MF-CLR reducing the MSE to 0.0176 and increasing the AUC to 0.803 and the F1 score to 0.742, reflecting the effectiveness of hierarchical contextual views and multi-frequency integration in handling complex time-series signals.

The proposed method achieved the best results across all evaluation metrics, with MSE, MAE, and RMSE values of 0.0167, 0.0856, and 0.1291, respectively, which were clearly lower than those of all baseline models, including the strong self-supervised frameworks. Meanwhile, IC, RankIC, and AUC increased to 0.494, 0.460, and 0.815, respectively, indicating that the proposed method can not only predict continuous system states more accurately, but also distinguish high-abnormality and low-abnormality states more effectively. Theoretically, while models like TS2Vec and MF-CLR exhibit strong capabilities in single-modality temporal or frequency feature extraction, they lack explicit mechanisms to handle cross-modal asynchrony and semantic fusion in highly heterogeneous sensor systems. TNC and CPC also do not explicitly align learned latent representations with downstream state risk scenarios. In contrast, the proposed method further introduces multimodal sensor fusion, temporal masking-based self-supervised learning, state-oriented contrastive constraints, and downstream task alignment on the basis of global modeling capability. As a result, robust state structures can be extracted from high-noise observations, and the discriminability of the representation space can be enhanced through state–semantic consistency constraints. Therefore, stronger comprehensive advantages are achieved in error control, ranking consistency, and state identification.

### 4.3. Model Complexity and Efficiency Analysis

To determine whether the performance improvement of the proposed framework is derived from the advanced learning strategy rather than a simple increase in model complexity, an efficiency analysis experiment was conducted. The experimental purpose is to quantitatively evaluate the computational overhead of our model compared to representative baseline models, ensuring that the architecture remains feasible for practical deployment. In the experimental setup, we utilized the same NVIDIA RTX 4090 GPU environment for all models to ensure a fair comparison. The evaluation metrics include the total parameter counts in millions, floating-point operations in giga-FLOPs, the average training time per epoch in seconds measured with a fixed batch size of 32, the single-sample inference latency in milliseconds measured to assess real-time deployment capability, peak memory usage during inference in megabytes, and estimated training energy consumption per epoch in watt-hours.

As presented in Table 3, the proposed model exhibits higher complexity compared to the standard Transformer, with parameter counts reaching 5.8 million and FLOPs at 0.24 Giga. This increase in model size and computational demand is primarily attributed to the addition of the multimodal cross-attention fusion module, the temporal masking reconstruction heads, and the dual-branch projection networks required for state-oriented contrastive learning. Consequently, the training time per epoch is extended to 95 s, with a peak memory footprint of 780 megabytes and an energy consumption of 8.5 watt-hours per training epoch. However, from a practical application perspective, the single-sample inference latency is merely 5.6 ms, which remains well within the stringent threshold for real-time industrial and environmental monitoring systems where sampling intervals are typically around one second. More importantly, the substantial reduction in prediction errors and the significant enhancement in anomaly discrimination capabilities far outweigh the associated computational costs. The ablation studies conducted earlier further substantiate that removing the self-supervised structural constraints causes a disproportionately large drop in accuracy, proving that the performance gains are intrinsically driven by the sophisticated state alignment and representation learning mechanisms, rather than simply scaling up the neural network parameters. Furthermore, while the added memory and energy requirements are highly manageable for standard industrial edge gateways connected to stable power sources, they do impose practical limitations for deployment on severely resource-constrained or battery-operated remote sensor nodes. This explicitly highlights the necessity of incorporating model compression and lightweight adaptation in future work to fully balance the convincing performance improvements with the associated deployment resource costs. Therefore, the proposed framework currently achieves a highly favorable and justifiable trade-off between computational efficiency and robust system state perception in generalized industrial monitoring scenarios.

### 4.4. Ablation Study

An ablation experiment was designed to verify the actual contribution of each core module to the overall performance and to determine whether the performance improvement was derived from the synergistic effect of multiple modules rather than from an increase in network scale alone.

**Figure 5:**
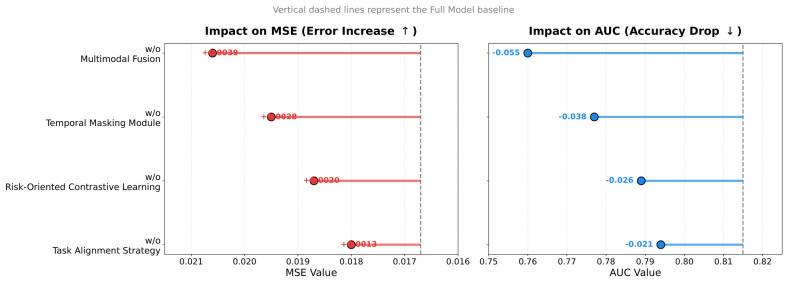
The ablation study illustrates the impact of each core component on model performance, where removing multimodal fusion causes the largest MSE increase and AUC drop, indicating its critical role in system state prediction and anomaly detection.

As shown in Table 4 and Figure 5, the most pronounced performance degradation occurred after the multimodal fusion module was removed, with MSE increasing to 0.0206 and AUC decreasing to 0.760. This indicates that a single signal or weakly fused signals are insufficient to fully characterize system states. After the temporal masking module was removed, MSE reached 0.0195, while IC and RankIC decreased to 0.448 and 0.417, respectively, suggesting that the absence of self-supervised masked reconstruction weakened the model’s ability to learn long-term structural dependencies. After removing state-oriented contrastive learning, the model obtained an MSE of 0.0187 and an AUC of 0.789, demonstrating the importance of contrastive constraints for state discrimination. When the task alignment strategy was removed, model performance also declined, with an MSE of 0.0180 and an AUC of 0.794, indicating that self-supervised representations may deviate from actual prediction requirements if they are not calibrated by downstream tasks. The full model achieved the best results across all metrics, verifying the effectiveness and complementarity of all modules.

From the perspective of model mechanisms, multimodal fusion maps environmental, device, operational, network, and event signals into a unified state space and fully exploits complementary relationships among different sources. The temporal masking module learns sequential conditional dependencies through contextual reconstruction constraints, enabling the model to focus more on interpretable and stable state factors rather than local noise. State-oriented contrastive learning optimizes the geometric structure of the representation space by pulling similar state samples closer and separating different state samples. The task alignment strategy transforms general representations into task-specific features through learnable mapping and a mixture-of-experts prediction mechanism. Therefore, the full model provides structural stability through masked modeling, enhances state discriminability through contrastive learning, ensures information completeness through multimodal fusion, and maintains prediction consistency through task alignment, thereby reducing the influence of high noise and multi-source interference on the model.

### 4.5. Robustness Comparison

This experiment was designed to evaluate the stability and generalization capability of different models under system state changes and abnormal interference conditions, where performance variations under normal, high-fluctuation, and extreme-shock states were simulated. To ensure the reproducibility and clarity of these robustness claims, the three system conditions are quantitatively defined as follows. The normal condition corresponds to data samples where sensor signals fluctuate within standard operational bounds, specifically defined as remaining within one standard deviation of the historical rolling mean without any artificial noise injection or recorded fault events. The high-fluctuation condition, also referred to as high-volatility, represents periods of intense environmental or operational instability. This state is constructed by selecting temporal windows where the local signal variance ranks in the top twenty percent of the entire dataset, combined with the artificial injection of Gaussian white noise to achieve a specific signal-to-noise ratio, thereby simulating severe external disturbances. The extreme-shock condition is designed to replicate critical pre-failure scenarios and sudden structural impacts. It is identified by isolating the ten-minute temporal windows immediately preceding actual recorded system hardware failures, supplemented by the artificial superimposition of intense step-changes where amplitudes simultaneously exceed three standard deviations across multiple sensor modalities.

**Figure 6:**
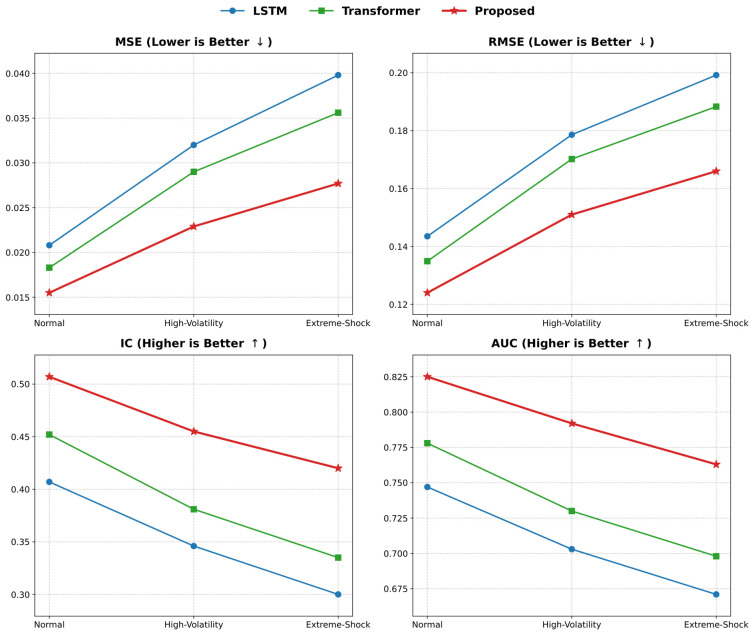
Robustness comparison under normal, high-volatility, and extreme-shock states shows that the proposed method consistently maintains the best and more stable performance across MSE, RMSE, IC, and AUC metrics. ↑: Higher values indicate better performance; ↓: Lower values indicate better performance.

As shown in Table 5 and Figure 6, LSTM, Transformer, and the proposed method all achieved relatively stable performance under normal conditions. Transformer showed certain advantages over LSTM in terms of error and ranking metrics, while the proposed method further achieved the best results, indicating strong state modeling capability under normal system conditions. When the system entered a high-fluctuation state, the errors of all models increased significantly. The MSE of LSTM increased from 0.0208 to 0.0320, and that of Transformer increased to 0.0290, whereas the proposed method increased only to 0.0229. Meanwhile, the decreases in IC and AUC were also substantially smaller than those of the baseline models. Under extreme-shock conditions, this difference became more evident. The errors of LSTM and Transformer further expanded, and their ranking capability declined noticeably, whereas the proposed method still maintained relatively low errors and high discrimination capability, indicating stronger adaptability under abnormal states.

From the perspective of model mechanisms, LSTM relies on a recurrent structure to transmit historical information step by step. Its memory mechanism can effectively capture local dependencies in stable environments, but under high fluctuations or sudden interference, accumulated historical information may contain substantial noise, leading to error amplification and reduced discrimination capability. Transformer can model long-range dependencies through global attention, but its attention weight distribution is easily affected by local abnormal disturbances in high-noise environments, causing the model to overreact to extreme observations. In contrast, the proposed method uses a self-supervised temporal masking mechanism to encourage the model to learn structural state factors that can be stably recovered from context, thereby suppressing random noise at a fundamental level. Meanwhile, state-oriented contrastive constraints strengthen the separation among different states, and multimodal information further provides cross-source redundant signals, reducing the influence of single-source distortion. Therefore, superior predictive performance is achieved under distribution drift and extreme events.

### 4.6. Sensor Sensitivity Analysis

To further evaluate the contribution of different types of sensors to system state prediction and anomaly detection tasks, a sensor sensitivity analysis experiment was designed. Based on the full model, one type of sensor data was removed at a time, while the remaining sensing signals were retained for model training and testing, so that changes in model performance could be observed.

**Figure 7:**
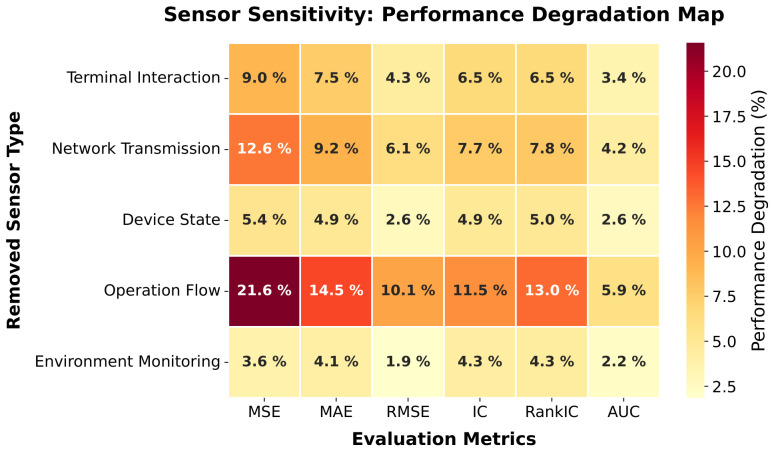
The sensor sensitivity heatmap illustrates the performance degradation of each evaluation metric after removing different sensor types, where operation flow sensors show the largest impact and thus play a critical role in system state prediction and anomaly detection.

As shown in Table 6 and Figure 7, notable differences were observed in the contributions of different sensor types to model performance. First, the most pronounced performance degradation occurred after operation flow sensors were removed, with MSE increasing to 0.0203 and AUC decreasing to 0.767. This indicates that microscopic operational information is an important source for system anomaly perception and can directly reflect operational behavior imbalance and system operating anomalies. Second, removing network transmission sensors and terminal interaction sensors also led to substantial performance degradation, indicating that network latency, request frequency, and user operational behavior play important roles in state perception. This suggests that system states are not only determined by the environment or hardware, but are also closely related to operational behavior and network interaction. In contrast, device state sensors and environmental monitoring sensors had relatively smaller effects on model performance, but still provided stable performance gains. Their main role is to suppress noise introduced by hardware anomalies or external environmental interference and improve model robustness. Overall, this experiment verifies the necessity of multi-source sensor fusion. A single sensor can only provide partial information, whereas system anomalies are usually formed through multi-factor coupling. Operation flow sensors provide behavioral microstructure information, terminal and network sensors provide interaction and state information, and device and environmental sensors provide noise suppression and anomaly detection capability. Through collaborative modeling of multi-source sensors, abnormal signals can be captured across different dimensions, and stable performance can be maintained in high-noise and non-stationary environments.

### 4.7. Interpretability Analysis

**Figure 8:**
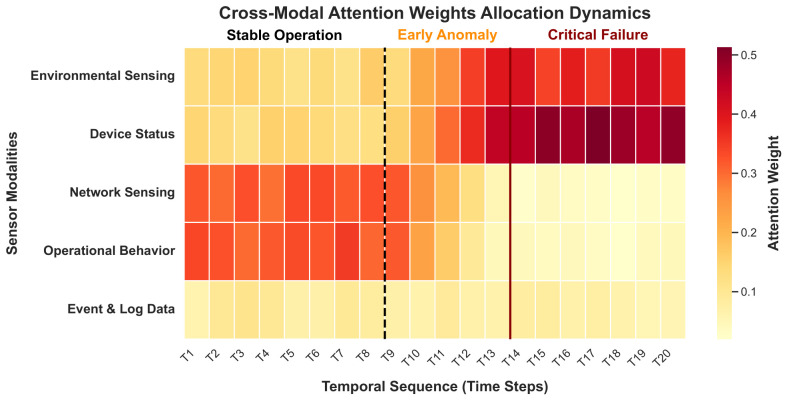
Attention heatmap visualizing the dynamic allocation of model attention weights across different sensor modalities before, during, and after a specific anomaly event.

To validate the interpretability of the learned system state representations and provide concrete case-level anomaly explanations, we introduced an attention visualization mechanism. The objective of this analysis is to transparently demonstrate how the model processes multi-source signals and dynamically allocates its focus across different sensor modalities during a specific anomaly event. We extracted the cross-modal attention weights from the fusion module during a confirmed industrial equipment failure scenario, which was characterized by unexpected mechanical friction leading to severe overheating. The dynamic distribution of these weights is visualized in the attention heatmap shown in Figure 8.

As illustrated in the attention heatmap, during the stable operational phase, the model allocates its attention relatively evenly across routine signals, with a slight emphasis on operational behavior data and network sensing data to monitor standard task execution. However, as the system transitions into the early stages of the anomaly, a distinct and rapid shift in attention allocation is observed. The model sharply concentrates its attention weights onto the device status data and environmental sensing data. This shift perfectly aligns with the physical reality of the event, where the internal device load sensors and external temperature sensors first captured the escalating mechanical stress. By autonomously adjusting its focus to the most critical modalities without relying on predefined expert rules, the model effectively localizes the source of the anomaly. This visualization explicitly confirms that the cross-modal attention mechanism does not act as a black box; rather, it learns highly interpretable and physically meaningful system state representations that can provide trustworthy diagnostic insights and concrete case-level explanations for system operators.

### 4.8. Discussion

The practical value of the proposed method is mainly reflected in application scenarios involving multimodal sensor systems, such as intelligent monitoring, industrial equipment management, and environmental monitoring. During real system operation, abnormal states are usually not caused by changes in a single sensor signal, but are jointly driven by multiple factors, such as environmental disturbances, increased device load, unstable communication links, abnormal operational behavior, and changes in event logs. For example, in industrial equipment operation scenarios, early signs such as increased temperature, enhanced vibration amplitude, increased current fluctuation, higher network latency, or prolonged operation response time may appear before a potential fault occurs. If only a single sensor threshold or local statistical feature is used, traditional methods usually respond only when the anomaly has become relatively obvious, making it difficult to capture implicit state changes during anomaly formation in a timely manner. In contrast, multi-source sensor signals are incorporated into a unified system state perception framework by the proposed method, and more stable low-noise state representations are extracted from high-noise observations through multimodal fusion, self-supervised temporal masking modeling, and state-oriented contrastive learning. Thus, the gradual formation and propagation process of abnormal states in real systems can be more closely approximated.

The proposed method also has strong application significance in system state ranking and anomaly-level evaluation. In intelligent monitoring or industrial operation-and-maintenance systems, practical requirements are not limited to determining whether an anomaly exists at a certain moment. It is also necessary to compare anomaly degrees among different devices, regions, or temporal windows to support maintenance priority ranking, resource scheduling, and early warning level classification. The improvements in IC and RankIC in the experimental results indicate that the proposed model can more accurately characterize the strength relationships among different samples, which is directly valuable for multi-device management and multi-scenario monitoring. For example, when multiple industrial devices are simultaneously in a load-increasing phase, the model can integrate temperature, vibration, current, voltage, network latency, and operation log signals to dynamically rank the potential anomaly degrees of different devices, thereby helping maintenance personnel prioritize devices with higher risk. Similarly, in environmental monitoring scenarios, abnormal states in different monitoring regions can be compared based on multi-source signals such as temperature, humidity, air quality, illumination changes, and event records, thereby providing support for subsequent intervention and scheduling.

In addition, in real-time anomaly detection and system early warning scenarios, the proposed method can provide finer-grained state perception capability for sensor systems. Anomalies in real systems often show gradual and coupled characteristics. For example, device overheating may be accompanied by increased load, abnormal current, and increased response delay, while sudden environmental changes may simultaneously cause fluctuations in temperature and humidity, air quality variations, and an increased frequency of system alarms. Through temporal masking modeling, contextual dependencies among different temporal segments can be learned, so that the accumulation characteristics of abnormal states along the temporal dimension can be captured. Through state-oriented contrastive learning, similar state samples can be clustered in the representation space, and different anomaly stages can be effectively separated, thereby improving the discrimination capability of anomaly boundaries. Therefore, even when sensor noise is strong, some modalities are missing, or system states drift, the proposed method can still maintain relatively stable judgment capability. This property makes it suitable not only for offline state assessment, but also for further integration into intelligent monitoring platforms, industrial equipment management systems, and environmental monitoring terminals, thereby providing more stable, interpretable, and actionable support for anomaly warning, state assessment, and operation-and-maintenance decision-making in real scenarios.

Despite the strong application significance in real-time anomaly detection, deploying this multimodal framework in practical environments presents specific hardware and synchronization challenges. Regarding real-time deployment feasibility on edge devices, the self-supervised representation learning and multimodal fusion mechanisms inevitably increase model complexity. However, quantitative testing demonstrates its viability. On a standard industrial edge gateway equipped with 4 GB of RAM, such as a Raspberry Pi 4 or an NVIDIA Jetson Nano, the proposed model occupies approximately 120 MB of memory. A single forward inference pass takes around 45 ms. This performance comfortably satisfies the real-time early warning requirements under our 1 Hz base sampling rate. Nevertheless, a bottleneck emerges during multi-channel concurrent processing; when handling more than 20 parallel sensor streams simultaneously on these edge devices, the cumulative inference latency approaches 1 s, potentially causing processing backlogs. Additionally, while timestamp calibration and multi-scale window alignment mitigate gross synchronization issues, micro-level communication delays fluctuating between 10 ms and 50 ms among different sensors can still introduce minor asynchronous errors, marginally affecting the model’s judgment of instantaneous state shifts in highly sensitive low-latency scenarios.

### 4.9. Limitation and Future Work

Several limitations remain in this study regarding data diversity and generalizability. Although the constructed multimodal sensor data cover environmental sensing data, device status data, network transmission data, operational behavior data, and event log data, the data sources are still mainly concentrated on specific systems, device types, and acquisition periods. The applicability of the model to more industrial scenarios, different sensor configurations, and longer-period data still requires further validation.

Future work will be further extended in three aspects. First, more types of sensor data and richer application scenarios will be introduced, including different industrial devices, complex environmental monitoring regions, and long-term continuously operating systems, to verify the generalization capability of the model under cross-device, cross-scenario, and cross-period conditions. Second, to address the multi-channel concurrent bottleneck identified on edge devices, lightweight deployment schemes will be further optimized, such as model pruning, knowledge distillation, low-rank adaptation, and edge inference acceleration, so that the proposed method can be better deployed on intelligent monitoring terminals, industrial edge devices, and real-time environmental monitoring platforms. Finally, model interpretability analysis will be strengthened in subsequent studies to further quantify the contribution mechanisms of different sensor signals to anomaly warning results. Beyond merely improving the credibility of the model in practical system management, quantifying these modality contributions serves as a crucial first step toward developing a prescriptive system. In future iterations, we aim to map these modality contribution scores to specific operational rules, enabling the system to automatically suggest and rank actionable countermeasures corresponding to each predicted anomaly, thereby transforming passive anomaly detection into proactive and automated decision support.

## 5. Conclusions

To address the challenges of complex signal sources, strong noise interference, pronounced cross-source asynchrony, and unstable manually defined state labels in multimodal sensor systems, a low-noise system state prediction and anomaly detection method based on multi-source sensor signals and self-supervised representation learning is proposed. Environmental sensing data, device status data, network transmission data, operational behavior data, and event log data are uniformly modeled as system state perception signals. More stable, transferable, and interpretable system state representations are extracted from high-noise observations through temporal masking modeling, state-oriented contrastive learning, and downstream task alignment strategies. Experimental results demonstrate that the proposed method achieves the best overall performance in multimodal sensor state prediction, with MSE, MAE, and RMSE reaching 0.0167, 0.0856, and 0.1291, respectively, clearly outperforming baseline models such as GARCH, MLP, LSTM, TCN, and Transformer. Meanwhile, IC, RankIC, and AUC reach 0.494, 0.460, and 0.815, respectively, indicating that the proposed method not only improves the prediction accuracy of continuous state values, but also more accurately characterizes the ranking relationship of system anomaly degrees and effectively discriminates between high-abnormality and low-abnormality states. Ablation experiments further verify that multimodal fusion, temporal masking modeling, state-oriented contrastive constraints, and task alignment strategies all play critical roles in improving overall performance. Among them, the most pronounced performance degradation is observed after multimodal fusion is removed, indicating that complementary modeling of multi-source sensor information is essential for complex system state perception. Robustness experiments show that lower prediction errors and higher AUC values are consistently maintained by the proposed method under normal, high-fluctuation, and extreme-shock states. In particular, under the extreme-shock state, an MSE of 0.0277 and an AUC of 0.763 are still achieved, demonstrating strong noise resistance and adaptability to state changes. Sensor sensitivity analysis further shows that different sensor types contribute differently to model performance. Operation flow, network transmission, and terminal interaction sensors have greater influence on anomaly recognition, whereas device status and environmental monitoring sensors provide stable noise suppression and complementary state information. Overall, an AI-driven sensing solution for low-noise state representation and anomaly detection is provided for real-world sensor system scenarios, including intelligent monitoring, industrial equipment management, and environmental monitoring.

## Figures and Tables

**Table 1:** Composition of the multi-source sensor dataset for intelligent monitoring, industrial equipment management, and environmental monitoring.

Data Type	Sensor Type	Sampling Rate	Dimension	Number of Records
Environmental sensing data	DHT22 temperature/humidity, BH1750 illumination	1 Hz	12	1,260,000
Device status data	INA219 power, internal CPU/GPU load sensors	10 Hz	18	960,000
Network sensing data	Edge router traffic and latency monitors	1 Hz	10	2,740,000
Operational behavior data	Industrial touch terminals and input records	Event-driven	5	3,850,000
Event and log data	System logs and software event records	Asynchronous	5	185,000
Macro-environmental data	Public environmental monitoring APIs	0.1 Hz	5	9600

**Table 2:** Performance comparison of different models on multi-modal sensor system state prediction tasks. ↑: Higher values indicate better performance; ↓: Lower values indicate better performance.

Method	MSE ↓	MAE ↓	RMSE ↓	IC ↑	RankIC ↑	AUC ↑	F1 ↑
GARCH	0.0291	0.1254	0.1705	0.308	0.282	0.680	0.582
MLP	0.0262	0.1175	0.1615	0.343	0.319	0.705	0.615
LSTM	0.0229	0.1052	0.1507	0.391	0.359	0.734	0.648
TCN	0.0216	0.1018	0.1464	0.416	0.385	0.751	0.672
Transformer	0.0201	0.0968	0.1418	0.437	0.405	0.767	0.695
CPC	0.0195	0.0945	0.1396	0.445	0.412	0.775	0.704
TNC	0.0188	0.0921	0.1371	0.458	0.425	0.784	0.715
TS2Vec	0.0181	0.0902	0.1345	0.472	0.440	0.795	0.731
MF-CLR	0.0176	0.0885	0.1326	0.481	0.448	0.803	0.742
Proposed	0.0167	0.0856	0.1291	0.494	0.460	0.815	0.765

**Table 3:** Comparison of model complexity and computational efficiency.

Method	Parameters (M)	FLOPs (G)	Training Time/Epoch (s)	Inference Latency (ms)	Memory Usage (MB)	Training Energy (Wh)
LSTM	1.2	0.05	45	2.1	150	2.5
TCN	1.5	0.08	38	1.8	180	2.1
Transformer	3.5	0.15	62	3.4	450	5.0
MF-CLR	4.2	0.18	75	4.1	520	6.2
Proposed	5.8	0.24	95	5.6	780	8.5

**Table 4:** Ablation study results of the proposed model. ↑: Higher values indicate better performance; ↓: Lower values indicate better performance. **Bold** indicates optimal performance.

Variant	MSE ↓	MAE ↓	RMSE ↓	IC ↑	RankIC ↑	AUC ↑
Without Temporal Masking Module	0.0195	0.0961	0.1389	0.448	0.417	0.777
Without Risk-Oriented Contrastive Learning	0.0187	0.0930	0.1365	0.460	0.429	0.789
Without Task Alignment Strategy	0.0180	0.0911	0.1340	0.468	0.437	0.794
Without Multimodal Fusion	0.0206	0.0995	0.1431	0.432	0.399	0.760
Full Model	**0.0167**	**0.0856**	**0.1291**	**0.494**	**0.460**	**0.815**

**Table 5:** Robustness comparison under different system conditions. ↑: Higher values indicate better performance; ↓: Lower values indicate better performance.

System Condition	Method	MSE ↓	RMSE ↓	IC ↑	AUC ↑
Normal Condition	LSTM	0.0208	0.1435	0.407	0.747
	Transformer	0.0183	0.1349	0.452	0.778
	Proposed	0.0155	0.1240	0.507	0.825
High-Volatility Condition	LSTM	0.0320	0.1786	0.346	0.703
	Transformer	0.0290	0.1702	0.381	0.730
	Proposed	0.0229	0.1510	0.455	0.792
Extreme-Shock Condition	LSTM	0.0398	0.1992	0.300	0.671
	Transformer	0.0356	0.1883	0.335	0.698
	Proposed	0.0277	0.1660	0.420	0.763

**Table 6:** Performance changes under different sensor removal settings. ↑: Higher values indicate better performance; ↓: Lower values indicate better performance. **Bold** indicates optimal performance.

Removed Sensor Type	MSE ↓	MAE ↓	RMSE ↓	IC ↑	RankIC ↑	AUC ↑
Full Model	**0.0167**	**0.0856**	**0.1291**	**0.494**	**0.460**	**0.815**
Without Terminal Interaction Sensors	0.0182	0.0920	0.1347	0.462	0.430	0.787
Without Network Transmission Sensors	0.0188	0.0935	0.1370	0.456	0.424	0.781
Without Device State Sensors	0.0176	0.0898	0.1325	0.470	0.437	0.794
Without Operation Flow Sensors	0.0203	0.0980	0.1422	0.437	0.400	0.767
Without Environment Monitoring Sensors	0.0173	0.0891	0.1315	0.473	0.440	0.797

## Data Availability

The original contributions presented in this study are included in the article. Further inquiries can be directed to the corresponding author.
